# P-1937. Multisystem inflammatory syndrome in children (MIS-C) - Accurate Diagnosis for Targeted Treatment

**DOI:** 10.1093/ofid/ofae631.2096

**Published:** 2025-01-29

**Authors:** Sanjana Sundararajan, Swetha Pinninti, Suresh Boppana

**Affiliations:** University of Alabama at Birmingham, Birmingham, Alabama; Heersink School of Medicine/University of Alabama at Birmingham, Birmingham, Alabama; University of Alabama at Birmingham, Birmingham, Alabama

## Abstract

**Background:**

Multisystem inflammatory syndrome in children (MIS-C) is a hyperinflammatory post-acute COVID-19 condition that clinically overlaps with Kawasaki disease (KD). However, the recommended first-line therapy differs for these two conditions. The Centers for Disease Control (CDC) and centers for state and territorial epidemiologists (CSTE) updated the surveillance case definition for MIS-C in 2023 to improve specificity. Since it has been suggested that 15%-20% of children identified using the 2020 definition will not meet the updated definition. While the incidence of MIS-C has decreased, waning immunity and low vaccine uptake in children requires continued vigilance and research. The objective of this study is to determine the utility of the 2023 CSTE/CDC definition to identify children with MIS-C at a quaternary care pediatric center.

**Figure:**
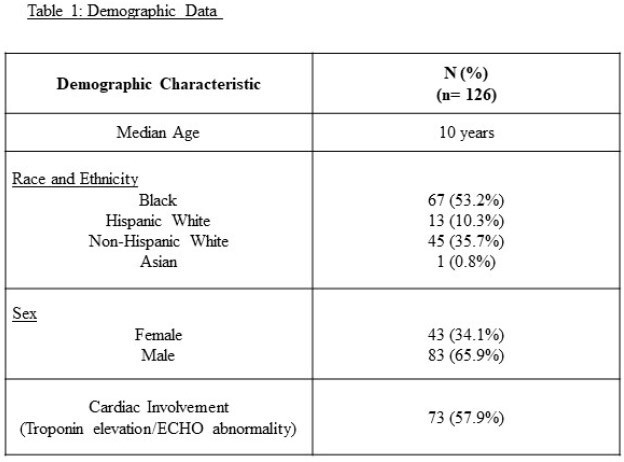

**Methods:**

Children diagnosed and treated for MIS-C at the Children’s of Alabama (COA) between January 2020 and December 2022 were enrolled in a prospective follow-up study. Demographic, clinical, and laboratory data, and treatment regimens were abstracted from the electronic medical record (EMR). The CDC 2020 and the 2023 CTSE/CDC criteria were applied to the cohort.

**Results:**

The study cohort includes 126 children diagnosed and treated for MIS-C at COA between 2020 and 2022. The demographic and clinical features are summarized in Table 1. Of the 126 children, 92.8% (117/126) and 93.6% (118/126) respectively met the 2020 and the updated 2023 definition for MIS-C. While 81.7% (103/126) of children were treated with IVIG + steroids, 18% (23/126) were treated with IVIG only. In addition, 12 children required immunomodulators (Anakinra /Remicade /Infliximab) for resolution of symptoms after treatment with IVIG + steroids. Favorable short and mid-term outcomes were documented in all children in this cohort.

**Conclusion:**

Over 90% of children with MIS-C met the criteria for MIS-C diagnosis with either the 2020 or the revised 2023 definition. The lack of definitive clinical or laboratory biomarkers and significant clinical overlap with other entities makes it challenging to identify all children with MIS-C highlighting the need for a better understanding of the pathogenesis, biomarker discovery, and effective therapies.

**Disclosures:**

Swetha Pinninti, MD, Moderna: Grant/Research Support|Pfizer: Grant/Research Support Suresh Boppana, MD, GSK: Advisor/Consultant|Merck: Grant/Research Support|Pfizer: Grant/Research Support

